# Factors affecting online health community participation behavior in patients with thyroid cancer

**DOI:** 10.1371/journal.pone.0235056

**Published:** 2020-06-24

**Authors:** Kyung Ah Park, So Yeon Eum, Hyeonjung Oh, Myung Hae Cho, Hang-Seok Chang, Yong Sang Lee, Sanghee Kim, Cheong Soo Park

**Affiliations:** 1 Thyroid Cancer Center Gangnam Severance Hospital, Yonsei University Health System, Seoul, Korea; 2 Division of Nursing, Gangnam Severance Hospital, Yonsei University Health System, Seoul, Korea; 3 Department of Nursing, Graduate School, Yonsei University, Seoul, Korea; 4 Department of Surgery, Yonsei University College of Medicine, Seoul, Korea; 5 Institute of Refractory Thyroid Cancer, Yonsei University, Seoul, Korea; 6 College of Nursing & Mo-Im Kim Nursing Research Institute, Yonsei University, Seoul, Korea; Fukushima Medical University School of Medicine, JAPAN

## Abstract

Globally, cancer patients obtain much of their disease information online. Online health communities allow patients to share questions and information about diseases. However, there have been few studies on the factors affecting online health community participation behavior in cancer patients. Online social networking is associated with mental health problems, and patients with thyroid cancer experience high levels of distress, anxiety and depression. The purpose of this study was to investigate factors associated with use of online health communities by patients with thyroid cancer to understand the characteristics of patients participating in such online communities. A questionnaire survey was completed by 114 thyroid cancer patients admitted for surgery at a general hospital in Seoul, Korea. General characteristics, clinical characteristics, attitude toward cancer, distress, and anxiety and depression scores of patients who joined an online health community (user group) and patients who did not (non-user group) were compared. The factors affecting online health community participation were education (*p* = 0.049), tumor size (*p* = 0.010), attitude toward cancer (*p* = 0.022), and anxiety and depression (*p* = 0.021). The average score of satisfaction with the online health community was 4.25 of 5. The user group had larger tumors, a high awareness of the risk of thyroid cancer, and high levels of anxiety and depression. Patients who actively used the online health community have relatively larger cancer size and had higher levels of mental stress. As such patients are often very anxious and depend heavily on the gathered information, the quality of this information is important. Healthcare professionals need to develop appropriate interventions for patients participating in the online health community.

## Introduction

The population of internet users continues to increase globally, and health care services are also becoming more internet-centric [1]. Patients who want to know about diseases usually obtain information from the internet [2]. The Internet has become an important medium for sharing health information and has also affected patients’ treatment decisions [3]. As more people connect through internet networks, hospitals are offering websites to provide medical information. However, as most of the hospital sites provide limited information, it is difficult for patients to find details such as costs and waiting times [4,5].

Online health communities offer a virtual environment in which patients with common interests freely communicate and share information and knowledge [6]. Patients can easily get support from peers through online health community [6,7]. Therefore, many patients participate in an online health community of others with shared experiences, and healthcare professionals want to provide services using an online health community. However, to provide online services for patients, it is necessary to know what kind of people are using online health communities. People have different interests and preferences, and internet use differs according to sex, age, and region [8]. Internet service providers are building big data based on user behavior in various areas and are using big data to make various customized advertisements [9]. Healthcare professionals will also be able to plan appropriate interventions for online users if they are aware of the characteristics of patients who are joining and using online communities.

In previous studies, online social networking has been associated with several mental illnesses such as depression and anxiety, as well as with low self-esteem [10,11]. Whether mental illness is causing online social networking or whether social online networking is causing mental illness is unclear. However, people using online social networking are emotionally vulnerable, and patients who join an online health community can be predicted to be emotionally vulnerable. Research on online health communities shows that the internet is an important tool for emotional intervention, and internet-mediated interventions have a positive effect on depression and anxiety [12–15]. However, few studies have focused on the characteristics of online health community users.

With the development of diagnostic technology worldwide, the incidence of thyroid cancer has been steadily increasing due to increased detection [16,17]. In Korea, thyroid cancer detection has increased rapidly and has shown the highest cancer incidence since 2009 [18]. Thyroid cancer is often described as a good cancer because it has a good prognosis and a good survival rate [19]. However, patients with thyroid cancer felt negative feelings and isolation about the fact that thyroid cancer represented as good cancer [19]. Patients with thyroid cancer experience high levels of distress and worry, despite having a good prognosis, and have more prevalent anxiety and depression than other cancers [20–22]. Cancer patients feel psychological pain such as anxiety and depression and seek social support [23]. Analyzing the characteristics of subscribers of a thyroid cancer community will help identify the mental health and needs of thyroid cancer patients.

The purpose of this study was to investigate factors associated with use of online health communities by patients with thyroid cancer to understand the characteristics of such patients participating in an online health community. In this study, we compared the characteristics, attitude toward cancer, distress, depression, and anxiety of online-community user and non-user groups. We also described the degree of online-community activity of the online-community user group and their satisfaction with the online community.

## Materials and methods

### Setting and participants

This study compares the characteristics of online health community user and non-user groups. The online health community in this study refers to the ‘Thyroid Family,’ established in April 2012 at the internet portal site NAVER [24]. The ‘Thyroid family’ was established in Naver, Korea’s largest portal site. The number of members was about 15,000, and the highest online community activity score among thyroid cancer-related online communities in Naver was selected for this study. The ‘Thyroid Family’ is operated by Gangnam Severance Medical Team for patients with thyroid cancer. The online health community consists of professional opinion columns, online booklets, patient experiences, and a Q&A board. Patients were divided according to subscription to the online health community. Those who joined the online health community were classified in the user group, and those who did not join the online health community were classified in the non-user group.

A questionnaire survey was conducted with 114 thyroid cancer patients aged 18 years or older, admitted for surgery from September 18 to November 13, 2017 at Gangnam Severance Hospital, Yonsei University Health System, Korea. To be eligible to participate, respondents required the ability to read and respond to questionnaires. A questionnaire was given to 120 patients admitted to surgery for thyroid cancer. Six patients who refused to participate in the study were excluded. All patients were informed of the guidelines for online communities, and exposure times may vary depending on the individual’s internet search and online use. A survey was conducted researcher knowledge of patient enrollment in an online community. There was no compensation for patient participation in the study. The questionnaire was completed before surgery for thyroid cancer.

This study was approved by the Institutional Review Board of Yonsei University Gangnam Severance Hospital (IRB No. 2017-0368-002). Participants were informed that participation was voluntary and were asked to complete the questionnaire in private. Participants provided written informed consent and contact details (email address and telephone number). Data were coded, and safety monitoring was performed every six months.

### Survey instrument

The questionnaire consisted of 33 items, including general characteristics, clinical characteristics, characteristics of the online health community, satisfaction with the online health community, attitude toward cancer, distress, and anxiety and depression. Standardized questionnaire tools were used to measure attitude toward cancer, distress, and the HADS (Hospital Anxiety and Depression Scale) score.

Characteristics of the online health community consisted of the reason for joining the community, the time of joining the community, the degree of community activity, and the level of satisfaction with the community. The measurement tool for assessing quality characteristics that affect satisfaction consisted of nine items and was designed by An et al. (2007) for use in a company internet community site survey [25]. The items addressed by the tool were convenience, reliability of information, usefulness, service playability, site type property, brand reputation, interactivity, satisfaction, and willingness to use in the future. Scores were evaluated using a five-point Likert scale where a higher score indicated greater satisfaction. Cronbach’s α = 0.927 indicated the reliability of the satisfaction evaluation tool.

The measurement tool used to measure attitude toward thyroid cancer was based on a survey tool developed by Suh et al. (1998) and modified by specialists, including one medical specialist and four nurses [26]. The tool consisted of four items related to general attitude toward cancer and six items related to attitude toward early detection and prevention of cancer. Scores were evaluated using a five-point Likert scale where a higher score indicated that the responder was more aware of the risk of thyroid cancer and was positive toward early detection and prevention of thyroid cancer. Cronbach’s α = 0.809 indicated the reliability of the tool to measure attitude toward thyroid cancer.

Distress Thermometer were developed to efficiently measure psychosocial distress and have proven to be an effective tool for screening for cancer-related distress [27, 28]. The Korean version of the Distress Thermometer was used in this study. The degree of psychological distress experienced by patients during the previous week was reported on a visual analogue scale from 0–10, with 0 being "no distress" and 10 being "extreme distress" [29]. Distress scores ≥4 means severe stress, so all patients with distress scores ≥4 were recommended psychological counseling after discharge.

The Hospital Anxiety and Depression Scale (HADS) is a tool for assessing the severity and symptoms of anxiety and depression and offers high sensitivity and specificity [30]. The Hospital Anxiety and Depression Scale (HADS) consists of 14 items, with one subscale of seven items measuring depression (HAD-D) and one subscale of seven items measuring anxiety (HAD-A). Each question consists of four points from 0–3 depending on the severity of the symptoms. The higher the score, the more severe the symptoms [31]. Cronbach’s α = 0.906 indicated the reliability of the tool to measure The Hospital Anxiety and Depression Scale. All patients with HADS scores **≥11** received recommended psychological counseling after discharge.

### Data analysis

The analysis was done using the IBM Statistical Package for Social Sciences (SPSS) Statistics version 25.0. Differences according to general characteristics and disease-related characteristics of the experimental and control groups were analyzed using the χ2 test, Fisher’s exact test, and linear by linear association test. Independent t-tests were used to investigate differences in attitude toward thyroid cancer, distress, and anxiety and depression scores. Logistic regression analysis was used to determine the actual factors affecting online health community usage.

## Results

### Patient characteristics

General characteristics of the 114 patients were as follows: 66.7% were female, age ranged from 18–73 years, the most common age group was 30–39 years (42.1%), the majority were college graduates (78.1%), and the majority had a spouse (82.5%). Most patients had papillary carcinoma (96.5%), 49.1% underwent less than total thyroidectomy, and 56.1% had a tumor size less than 1 cm in diameter. Moreover, 14.0% had direct family members with a history of thyroid cancer. Of the 114 patients, 44 enrolled in an online health community (38.6%), and 93.2% of those enrolled used the online health community as an information source (Table 1).

**Table 1:** Characteristics of online health community users and non-users (n = 114).

Variables	Category	Online health community n (%)		Total	*p*
		User (n = 44)	Non-User (n = 70)		
**Age (years)**					
	Under 29	2 (4.5%)	7 (10.0%)	9 (7.9%)	
	30–39	24 (54.5%)	24 (34.3%)	48 (42.1%)	
	40–49	16 (36.4%)	12 (17.1%)	28 (24.6%)	
	Over 50	2 (4.5%)	27 (38.6%)	29 (25.4%)	.017
**Sex**					
	Male	9 (20.5%)	29 (41.4%)	38 (33.3%)	
	Female	35 (79.5%)	41 (58.6%)	76 (66.7%)	.021
**Married**					
	Yes	40 (90.9%)	54 (77.1%)	94 (82.5%)	
	No	4 (9.1%)	16 (22.9%)	20 (17.5%)	.078
**Education**					
	≤High school graduate	3 (6.8%)	22 (31.4%)	25 (21.9%)	
	≥College graduate	41 (93.2%)	48 (68.6%)	89 (78.1%)	.002
**Family History of Thyroid Cancer**					
	Yes	6 (13.6%)	10 (14.3%)	16 (14.0%)	
	No	38 (86.4%)	60 (85.7%)	98 (86.0%)	.923
**Information Source**					
	Online Community	41 (93.2%)	7 (10.1%)	48 (42.5%)	
	Other^a^	3 (6.8%)	62 (89.9%)	65 (57.5%)	.000
**Cancer Type**					
	Papillary	41 (93.2%)	69 (98.6%)	110 (96.5%)	
	Other^b^	3 (6.8%)	1 (1.4%)	4 (3.5%)	.297
**Extent of Surgery**					
	Less-than-total^c^	23 (52.3%)	33 (47.1%)	56 (49.1%)	
	Total thyroidectomy	17 (38.6%)	35 (50.0%)	52 (45.6%)	
	Other^d^	4 (9.1%)	2 (2.9%)	6 (5.3%)	.923
**Cancer Size**					
	< 1cm	19 (43.2%)	45 (64.3%)	64 (56.1%)	
	≥1cm	25 (56.8%)	25 (35.7%)	50 (43.9%)	.027

^**a**^ Other includes medical staff, internet surfing, books, TV, and people

^**b**^ Other includes Hurthle cell cancer and follicular cancer

^**c**^ Less-than-total includes less-than-total thyroidectomy and hemithyroidectomy

^**d**^ Other includes total thyroidectomy along with lateral neck dissection and re-operation

### Characteristics of the online health community-user group

Most subjects in the online health community-user group were registered in only one thyroid cancer online health community (65.9%), and participation time was highest two months before surgery (43.2%). The largest number of respondents found the online health community through an internet search (47.7%), and half (50.0%) of respondents said that they accessed the community more than once a day (Table 2).

**Table 2:** Characteristics of patients using an online health community (n = 44).

Variables	Category	n (%)
Number of online health community subscriptions related to thyroid cancer	1	29 (65.9%)
	2–3	15 (34.1%)
When the online health community ‘Thyroid Family’ was joined	2 months prior	19 (43.2%)
	2 month– 1 month	13 (29.5%)
	1 month– 1 week prior	8 (18.2%)
	1 week–today	4 (9.1%)
Motivation to join the online health community ‘Thyroid family’	Internet search	21 (47.7%)
	Hospital information	18 (40.9%)
	Introduced by a friend	5 (11.4%)
Frequency of accessing the online health community ‘Thyroid Family’	More than once a day	22 (50.0%)
	2–3 times a week	12 (27.3%)
	Once a week	8 (18.2%)
	Almost never	2 (4.5%)

### Attitude toward thyroid cancer, distress, and HADS

The online health community-user group was more aware of risk of thyroid cancer but also had a positive attitude toward early detection and prevention of the thyroid cancer. The online health community-user group had higher depression and anxiety scores and distress levels (Table 3). The HADS depression mean score of the user group was 8.09 and the HADS depression mean score of non-user group was 5.70 (*p* = 0.002), and the HADS anxiety mean score of the user group was 8.95 and the HADS anxiety mean score of non-user group was 6.79 (*p* = 0.004).

**Table 3:** Attitude, distress & HADS scores of online health community users and non-users (n = 114).

Attitude, Distress, HADS Score	Online health community (Mean±SD)		*F*	*p*
	User (n = 44)	Non-User (n = 70)		
**Attitude toward Thyroid Cancer ①–④**	15.55±2.54	13.63±2.46	0.085	.000
① Thyroid cancer is a serious disease.	3.70±0.70	3.14±0.80	0.647	.000
② I think thyroid cancer patients are the same as other cancer patients.	4.11±0.69	3.70±0.82	1.549	.006
③ I think thyroid cancer will hinder your home and work life.	3.82±0.92	3.36±0.84	0.081	.007
④ I’m afraid because I have thyroid cancer.	3.91±0.86	3.43±0.89	2.642	.005
**Attitude toward early detection and prevention of thyroid cancer ⑤–⑩**	27.14±2.43	25.56±2.59	0.012	.002
⑤ Screening for early detection of thyroid cancer is necessary.	4.50±0.59	4.29±0.75	0.415	.109
⑥ Early treatment can prevent cancer progression.	4.64±0.53	4.37±0.75	3.643	.043
⑦ I will practice cancer prevention in my daily life.	4.43±0.66	4.13±0.64	2.865	.016
⑧ I think that life can be better if thyroid cancer is discovered and treated early.	4.45±0.55	4.39±0.57	0.025	.526
⑨ I want to know more about thyroid cancer.	4.45±0.59	4.10±0.68	0.559	.002
⑩ I would recommend the tests necessary for detection of thyroid cancer to my family	4.61±0.58	4.29±0.66	1.118	.008
**Distress Scale**	6.41±2.42	5.25±2.63	0.084	.020
**HADS**	17.05±7.36	12.49±6.77	0.409	.001
HADS Depression	8.09±4.14	5.70±3.82	0.460	.002
HADS Anxiety	8.95±3.99	6.79±3.71	1.410	.004

### Factors affecting online health community subscription

Factors influencing online health community usage were education, tumor size, attitude toward cancer, anxiety, and depression. Online health community subscription was 4.3 times more likely in ≥college graduates than ≤high school graduates (B = 1.464, *p* = 0.049). Patients with tumors greater than 1 cm in diameter used an online health community 4.1 times more those with tumors less than 1 cm in diameter (B = 1.415, *p* = 0.010). In addition, those with higher attitude scores for thyroid cancer (B = 0.162, *p* = 0.022) and higher HADS score were also more likely to use the online health community (B = 0.102, *p* = 0.021) (Table 4).

**Table 4:** Factors associated with use of an online health community (n = 114).

Variables	Category	B	SE	OR (95% CI)	*p*
Age		-0.021	0.028	0.979(0.927~1.035)	.461
Sex	Male			1.000	
	Female	0.869	0.567	2.386(0.785~7.254)	.125
Married	Yes			1.000	
	No	-1.409	0.828	0.244(0.048~1.237)	.089
Education	≤High school graduate			1.000	
	≥College graduate	1.464	0.743	4.324 (1.007~18.560)	.049
Extent of surgery	Less-than-total^a^			1.000	
	Others	-1.017	0.551	0.362(0.123~1.065)	.065
Cancer size	< 1cm			1.000	
	≥ 1cm	1.415	0.550	4.118 (1.401~12.106)	.010
Attitude toward thyroid cancer		0.162	0.071	1.176(1.024~1.350)	.022
Distress		-0.132	0.125	0.877(0.686~1.121)	.294
HADS score		0.102	0.044	1.107 (1.015~1.208)	.021

^**a**^ Less-than-total includes less-than-total thyroidectomy and hemithyroidectomy

### Online health community satisfaction

Satisfaction with the online health community among the online health community-user group was generally high, with an average of 4.25 points on a five-point Likert scale. The perceived usefulness of the online health community scored 4.48 points out of a possible five points, and the intention to continue use scored 4.39 out of a possible five points (Table 5).

**Table 5:** Patient satisfaction with using an online health community (n = 44).

Variables		Mean±SD	Rank
Usefulness	The online community helps me	4.48±0.69	1
Willingness to continuously use	I want to continuously use the online community	4.39±0.69	2
Interactivity	Share experiences and communicate with others	4.36±0.75	3
Satisfaction	I am satisfied with the ‘Thyroid Family’ community	4.34±0.78	4
Reliability of information	Provides accurate and reliable information	4.32±0.67	5
Convenience	Easy to use	4.27±0.69	6
Site type properties	Good configuration and content	4.18±0.78	7
Service playability	I enjoy using the online community	3.95±0.83	8
Brand reputation	The ‘Thyroid Family’ community is well known	3.95±0.89	8
**Total**		**38.25±5.36**	

## Discussion

The use of online health communities was affected by education, tumor size, attitude toward thyroid cancer, anxiety, and depression. Most patients in the online health community-user group were college education or higher. In previous surveys, 99% non-internet users had a high school education or less, and college education patients were almost 3 times more likely to use the internet than those with a high school education or less. [8, 32]. College educated people get a lot of information online, and they seem to be more likely to join the online health communities.

Thyroid papillary cancer is the most common of thyroid cancers, and papillary microcarcinoma of less than 1cm are low risk of metastasis and sometimes observation is recommended without immediate surgery [33]. However, if the size of the thyroid papillary cancer is more than 1cm, the possibility of lymph node metastasis increases [34]. Patients with tumor size greater than 1 cm had a higher rate of online health community participation, possibly due to the belief that their disease was more serious and frightening than those with tumor size less than 1 cm.

People with a positive attitude toward early detection and prevention were also more likely to join an online health community in response to a cancer diagnosis. Patients with a strong desire to promote health are more active in searching for disease-related information [35]. The online user group showed the highest levels of anxiety and depression. When diagnosed with cancer, patients are faced with treatment burdens and the possibility of death [36]. Thyroid cancer patients often experience anxiety and depression after diagnosis [37,38]. Newly diagnosed breast cancer patients are also more likely to develop anxiety and depression [39]. The patients in this study consisted of preoperative patients, and the online community-user group consisted of preoperative patients who were diagnosed with thyroid cancer and showed mild anxiety and depressive symptoms. Joining an online health community is one way to reduce anxiety and depression by gathering information and sharing experiences with others. The motivation factor for online health community activity is to gain information and make changes in response to a cancer diagnosis [40]. Previous study has shown that health-related internet use was associated with depression, and those who are more vulnerable to health anxiety can choose online health resources [11]. If medical professionals can select patients who are at high risk for anxiety and depression from an online health community before surgery, timely intervention can be achieved.

Online health communities are easily accessible via the internet, and members can exchange information and emotional support. However, because inaccurate information is also common in online health communities, credibility must be secured for sites providing medical information to be effective [41]. It is sometimes difficult for people to determine what is correct, because information is sometimes provided for commercial purposes. Moreover, emotionally unstable patients are easily tricked and often have difficulty in discerning accurate information. In previous research, the quality of a website for thyroid cancer was found to be very different depending on the provider of website, and most of the websites were of low quality [42]. If patients in critical condition join an online health community that is not properly managed, they may be misled with wrong or incomplete information. Therefore, it is important for medical staff to improve the reliability of online health communities and guide members to share correct information and communication. The online health community accessed by subjects of this study was operated by medical staff, and satisfaction with the site among the online health community-user group was also high. When people experience the benefits of community services, the frequency of use of services increases [43]. In this study, more than half of the online health community-user group visited the site more than once a day, which shows that patients were self-directed in looking for the online health community, and that the online health community had a significant impact on patients.

Participation in online health communities has been shown to have a positive effect on knowledge sharing, and the social support provided by internet communities has been shown to increase self-efficacy and self-care behaviors [44,45]. Peer-support not only helps patients cope effectively with stressful situations, but it can also reduce depression and improves health status [46,47]. Technology is continuously changing, and the methods of implementing interventions are becoming more diverse. The use of the internet has increased, and online communication between patients and healthcare providers has also increased [48]. More research is needed on the characteristics of cancer patients who join an online health community and how the online health community affects cancer patients. Through continuous efforts and research, the online health community should be able to broaden its scope as an appropriate intervention for patients.

## Limitation

There are several limitations of the study. First, because the study utilized a pre-established online health community called a ‘Thyroid Family,’ which has been managed by a university affiliated hospital, the community does not represent the online community in general. Second, the results of this study are difficult to be generalized to other online disease-related communities because the characteristics of health communities are widely varied by multiple pathophysiological and societal factors. Third, as a nature of an observational study based on questionnaire, it is hard to claim any causality in our observations. More research is necessary to investigate the characteristics of people who are actively involved in an online health community.

## Conclusion

This study compared the characteristics of thyroid cancer patients who joined the online health community and those who did not, and identified factors that influenced the online health community participation behavior. Patients who actively used the online health community have relatively larger cancer size and more severe anxiety and depression. Healthcare professionals need to develop appropriate interventions for patients participating in the online health community.
