# Correction: Factors affecting online health community participation behavior in patients with thyroid cancer

**DOI:** 10.1371/journal.pone.0238273

**Published:** 2020-08-20

**Authors:** Kyung Ah Park, So Yeon Eum, Hyeonjung Oh, Myung Hae Cho, Hang-Seok Chang, Yong Sang Lee, Sanghee Kim, Cheong Soo Park

The ORCID iD is missing for the seventh author. Author Sanghee Kim’s ORCID iD is: 0000-0002-9806-2757 (https://orcid.org/0000-0002-9806-2757).

## References

[1] Park KA, Eum SY, Oh H, Cho MH, Chang H-S, Lee YS, et al (2020) Factors affecting online health community participation behavior in patients with thyroid cancer. PLoS ONE 15(6): e0235056 10.1371/journal.pone.0235056 32579575 PMC7313971

